# Poly[[diaqua­(μ_2_-1,4-dioxane-κ^2^ *O*:*O*′)(μ_2_-2,3,5,6-tetra­fluoro­benzene-1,4-dicarboxyl­ato-κ^2^ *O* ^1^:*O* ^4^)copper(II)] 1,4-dioxane disolvate dihydrate]

**DOI:** 10.1107/S1600536811009755

**Published:** 2011-04-07

**Authors:** Jing Yu, Yi-Feng Zhang, Fu-An Sun, Qun Chen

**Affiliations:** a Key Laboratory of Fine Petrochemical Technology, Jiangsu Polytechnic University, Changzhou 213164, People’s Republic of China

## Abstract

In the title complex, {[Cu(C_8_F_4_O_4_)(C_4_H_8_O_2_)(H_2_O)_2_]·2C_4_H_8_O_2_·2H_2_O}_*n*_, the Cu^II^ ion is six-coordinated by two oxygen donors from two *trans* 2,3,5,6-tetra­fluoro-1,4-dicarboxyl­ate (BDC-F_4_) ligands, two O atoms from two chair 1,4-dioxane ligands and two O atoms from two terminal water mol­ecules, adopting a distorted octa­hedral coordinated geometry. Each BDC-F_4_ anion bridges two Cu^II^ ions in a bis-monodentate fashion, forming a [Cu(BDC-F_4_)]_*n*_ chain. These chains are further linked by bridging 1,4-dioxane ligands, generating a two-dimensional net with approximately recta­ngular grids of 11.253 × 7.654 Å. Such adjacent parallel layers are connected by O—H⋯O hydrogen bonds between guest water mol­ecules and the uncoordinated carboxyl­ate O atoms and coordinated water mol­ecules into the final three-dimensional supra­molecular network.

## Related literature

For the solvent template effect of 1,4-dioxane in the construction of coordination polymers, see: Chen *et al.* (2008 ▶); He *et al.* (2009 ▶).
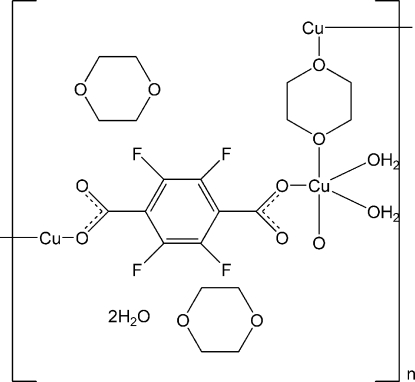


## Experimental

### 

#### Crystal data

[Cu(C_8_F_4_O_4_)(C_4_H_8_O_2_)(H_2_O)_2_]·2C_4_H_8_O_2_·2H_2_O
*M* *_r_* = 636.00
Monoclinic, 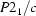

*a* = 7.654 (2) Å
*b* = 11.253 (3) Å
*c* = 16.126 (4) Å
β = 99.634 (6)°
*V* = 1369.4 (6) Å^3^
*Z* = 2
Mo *K*α radiation
μ = 0.89 mm^−1^
*T* = 297 K
0.20 × 0.15 × 0.12 mm

#### Data collection

Bruker APEXII CCD diffractometer
Absorption correction: multi-scan (*SADABS*; Sheldrick, 2003 ▶) *T*_min_ = 0.842, *T*_max_ = 0.901
7646 measured reflections
2536 independent reflections
2011 reflections with *I* > 2σ(*I*)
*R*_int_ = 0.030

#### Refinement

*R*[*F* ^2^ > 2σ(*F* ^2^)] = 0.052
*wR*(*F* ^2^) = 0.182
*S* = 1.07
2536 reflections
173 parameters
H-atom parameters constrained
Δρ_max_ = 0.77 e Å^−3^
Δρ_min_ = −0.47 e Å^−3^

### 

Data collection: *APEX2* (Bruker, 2007 ▶); cell refinement: *APEX2* and *SAINT* (Bruker, 2007 ▶); data reduction: *SAINT*; program(s) used to solve structure: *SHELXTL* (Sheldrick, 2008 ▶); program(s) used to refine structure: *SHELXTL*; molecular graphics: *SHELXTL* and *DIAMOND* (Brandenburg, 2005 ▶); software used to prepare material for publication: *SHELXTL*.

## Supplementary Material

Crystal structure: contains datablocks I, global. DOI: 10.1107/S1600536811009755/jj2078sup1.cif

Structure factors: contains datablocks I. DOI: 10.1107/S1600536811009755/jj2078Isup2.hkl

Additional supplementary materials:  crystallographic information; 3D view; checkCIF report

## Figures and Tables

**Table 1:** Hydrogen-bond geometry (Å, °)

*D*—H⋯*A*	*D*—H	H⋯*A*	*D*⋯*A*	*D*—H⋯*A*
O4—H4*B*⋯O7	0.82	1.92	2.670 (5)	152
O4—H4*A*⋯O1^i^	0.82	1.85	2.641 (3)	162
O7—H7*C*⋯O6^ii^	0.82	2.03	2.807 (7)	158
O7—H7*D*⋯O1^iii^	0.82	2.09	2.797 (5)	144

Symmetry codes: (i) 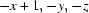; (ii) 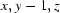; (iii) 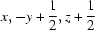.

**Fig. 1.**
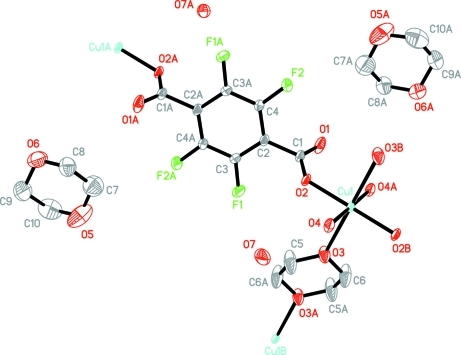
The molecular structure of the title compound, (I), with the atom-numbering scheme. Displacement ellipsoids are draw at the 30% probability level. Hydrogen atoms are omitted for clarity. Symmetry code: (A) 1 - x, 1 - y, -z; (B) 1 - x, -y, -z.

**Fig. 2.**
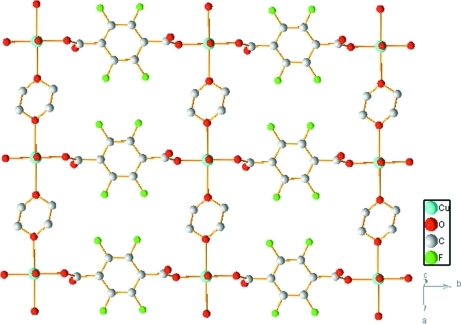
The two-dimensional structure of the title compound viewed down the c axis. Hydrogen atoms are omitted for clarity.

## supplementary crystallographic information

### Comment

Recently, our group has been engaged in studying the influence that a range of solvent species have on the structures of a series of Mn^II^—BDC-Cl_4_ polymers with 2,3,5,6-tetracholorobenzene-1,4-dicarboxylate (BDC-Cl_4_) ligand (Chen *et al.*, 2008; He *et al.*., 2009). Among them, the 1,4-dioxane (dioxane) solvent molecule may serve as a solvent template to play a key role in controlling the resulting polymeric network. To further understand the solvent template effect of 1,4-dioxane, we employed the tetrafluorinated benzene-1,4-dicarboxylic acid (H_2_BDC-F_4_) ligand to assemble with a copper(II) ion in the presence of dioxane and obtained the title two-dimensional coordination polymer {[Cu(BDC-F_4_)(dioxane)(H_2_O)_2_].(dioxane)_2_(H_2_O)_2_}*_n_*, (I).

The asymmetric unit of (I) is composed of one Cu^II^ center, one 2,3,5,6-tetrafluoro-1,4-dicarboxylate (BDC-F_4_) anion, one dioxane ligand, two coordinated water molecules, and two lattice dioxane as well as two water moieties (Fig. 1).  Each Cu^II^ ion is six-coordinated by two oxygen donors from two *trans* 2,3,5,6-tetrafluoro-1,4-dicarboxylate (BDC-F_4_) ligands, two oxygen atoms from two chair dioxane ligands, and two oxygen atoms from two terminal water molecules, adopting a distorted octahedral coordinated geometry.  Each BDC-F_4_ anion bridges two Cu^II^ ions in a bis-monodentate fashion to form a one-dimensional [Cu(BDC-F_4_)]*_n_* chain, which is further joined together by bridging dioxane ligands to generate a two-dimensional net with approximately rectangular grids of 11.253 Å × 7.654 Å (Cu···Cu nucleus-to-nucleus), where the Cu···Cu···Cu angles are 90.8 and 89.2°, respectively (Fig. 2). Such adjacent parallel layers are connected by O—H···O hydrogen bonds between guest water molecules with the uncoordinated carboxylate oxygen atoms and coordinated water molecules to fulfill the final three-dimensional supramolecular network.

### Experimental

An aqueous solution (2 ml) of Cu(ClO_4_)_2_.6H_2_O (37.1 mg, 0.10 mmol) was added to a dioxane solution (4 ml) of H_2_BDC-F_4_ (23.8 mg, 0.10 mmol) with stirring for 15 min. Then, the reaction mixture was filtered and left to stand at room temperature. After 3 days, well blue block crystals of the title compound suitable for X-ray diffraction were obtained by slow evaporation of the solvents in 52% yield (33.1 mg, based on H_2_BDC-F_4_).

### Refinement

All H atoms bound to C atoms were assigned to calculated positions with C—H = 0.97 Å, and refined using a riding model, with *U*_iso_(H)=1.2U_eq_ (C). The H atoms of the water molecule were firstly located in a difference Fourier map and then refined with distance restraints O—H = 0.820 (1) Å and H···H = 1.430 (1) Å, and finally constrained to ride on the O atom with [*U*_iso_(H) = 1.5 U_eq_ (O)].

### Crystal data

**Table:**

[Cu(C_8_F_4_O_4_)(C_4_H_8_O_2_)(H_2_O)_2_]·2C_4_H_8_O_2_·2H_2_O	*F*(000) = 658
*M**_r_* = 636.00	*D*_x_ = 1.543 Mg m^−^^3^
Monoclinic, *P*2_1_/*c*	Mo *K*α radiation, λ = 0.71073 Å
Hall symbol: -P 2ybc	Cell parameters from 3648 reflections
*a* = 7.654 (2) Å	θ = 2.6–29.9°
*b* = 11.253 (3) Å	µ = 0.89 mm^−^^1^
*c* = 16.126 (4) Å	*T* = 297 K
β = 99.634 (6)°	Block, blue
*V* = 1369.4 (6) Å^3^	0.20 × 0.15 × 0.12 mm
*Z* = 2	

### Data collection

**Table:**

Bruker APEXII CCD diffractometer	2536 independent reflections
Radiation source: fine-focus sealed tube	2011 reflections with *I* > 2σ(*I*)
graphite	*R*_int_ = 0.030
φ and ω scans	θ_max_ = 25.5°, θ_min_ = 2.2°
Absorption correction: multi-scan (*SADABS*; Sheldrick, 2003)	*h* = −9→9
*T*_min_ = 0.842, *T*_max_ = 0.901	*k* = −11→13
7646 measured reflections	*l* = −19→19

### Refinement

**Table:**

Refinement on *F*^2^	Primary atom site location: structure-invariant direct methods
Least-squares matrix: full	Secondary atom site location: difference Fourier map
*R*[*F*^2^ > 2σ(*F*^2^)] = 0.052	Hydrogen site location: inferred from neighbouring sites
*wR*(*F*^2^) = 0.182	H-atom parameters constrained
*S* = 1.07	*w* = 1/[σ^2^(*F*_o_^2^) + (0.1255*P*)^2^ + 0.7817*P*] where *P* = (*F*_o_^2^ + 2*F*_c_^2^)/3
2536 reflections	(Δ/σ)_max_ < 0.001
173 parameters	Δρ_max_ = 0.77 e Å^−^^3^
0 restraints	Δρ_min_ = −0.47 e Å^−^^3^

### Special details

**Table:**

Geometry. All e.s.d.'s (except the e.s.d. in the dihedral angle between two l.s. planes) are estimated using the full covariance matrix. The cell e.s.d.'s are taken into account individually in the estimation of e.s.d.'s in distances, angles and torsion angles; correlations between e.s.d.'s in cell parameters are only used when they are defined by crystal symmetry. An approximate (isotropic) treatment of cell e.s.d.'s is used for estimating e.s.d.'s involving l.s. planes.
Refinement. Refinement of *F*^2^ against ALL reflections. The weighted *R*-factor *wR* and goodness of fit *S* are based on *F*^2^, conventional *R*-factors *R* are based on *F*, with *F* set to zero for negative *F*^2^. The threshold expression of *F*^2^ > σ(*F*^2^) is used only for calculating *R*-factors(gt) *etc*. and is not relevant to the choice of reflections for refinement. *R*-factors based on *F*^2^ are statistically about twice as large as those based on *F*, and *R*- factors based on ALL data will be even larger.

### Fractional atomic coordinates and isotropic or equivalent isotropic displacement parameters (Å^2^)

**Table:**

	*x*	*y*	*z*	*U*_iso_*/*U*_eq_	
Cu1	0.5000	0.0000	0.0000	0.0256 (3)	
O1	0.4795 (5)	0.2295 (2)	−0.12878 (17)	0.0582 (9)	
O2	0.4927 (3)	0.1749 (2)	0.00527 (13)	0.0332 (6)	
O3	0.1848 (4)	−0.0053 (3)	0.0096 (3)	0.0592 (10)	
O4	0.5645 (4)	0.00298 (18)	0.12299 (16)	0.0356 (6)	
H4A	0.5557	−0.0669	0.1362	0.053*	
H4B	0.5186	0.0562	0.1466	0.053*	
O5	−0.0024 (8)	0.7388 (5)	0.2701 (5)	0.135 (2)	
O6	0.2054 (7)	0.9379 (4)	0.3007 (4)	0.1177 (19)	
O7	0.4024 (5)	0.1145 (4)	0.2350 (2)	0.0845 (13)	
H7C	0.3248	0.0770	0.2530	0.127*	
H7D	0.4640	0.1633	0.2643	0.127*	
C1	0.4872 (4)	0.2499 (3)	−0.0530 (2)	0.0317 (8)	
C2	0.4925 (4)	0.3790 (3)	−0.0259 (2)	0.0314 (7)	
C3	0.3590 (5)	0.4308 (3)	0.0086 (3)	0.0442 (10)	
C4	0.6337 (5)	0.4519 (4)	−0.0345 (3)	0.0431 (9)	
C5	0.1002 (7)	0.0757 (7)	0.0515 (6)	0.111 (3)	
H5A	0.1186	0.0516	0.1101	0.133*	
H5B	0.1612	0.1508	0.0492	0.133*	
C6	0.0753 (7)	−0.0976 (6)	−0.0285 (6)	0.101 (3)	
H6A	0.1210	−0.1224	−0.0784	0.121*	
H6B	0.0881	−0.1645	0.0099	0.121*	
C7	0.1634 (10)	0.7455 (7)	0.2473 (5)	0.109 (2)*	
H7A	0.1508	0.7722	0.1894	0.131*	
H7B	0.2155	0.6667	0.2503	0.131*	
C8	0.2799 (8)	0.8240 (5)	0.2992 (4)	0.0816 (16)	
H8A	0.3044	0.7925	0.3560	0.098*	
H8B	0.3912	0.8293	0.2782	0.098*	
C9	0.0421 (9)	0.9318 (7)	0.3302 (6)	0.110 (3)	
H9A	−0.0091	1.0105	0.3307	0.132*	
H9B	0.0606	0.9009	0.3872	0.132*	
C10	−0.0808 (8)	0.8519 (9)	0.2736 (5)	0.111 (3)	
H10A	−0.1918	0.8443	0.2946	0.133*	
H10B	−0.1058	0.8859	0.2176	0.133*	
F1	0.2156 (4)	0.3659 (2)	0.0169 (3)	0.0886 (12)	
F2	0.7708 (4)	0.4067 (2)	−0.0662 (2)	0.0849 (12)	

### Atomic displacement parameters (Å^2^)

**Table:**

	*U*^11^	*U*^22^	*U*^33^	*U*^12^	*U*^13^	*U*^23^
Cu1	0.0368 (4)	0.0138 (4)	0.0265 (4)	0.0004 (2)	0.0061 (2)	−0.00102 (18)
O1	0.118 (3)	0.0239 (14)	0.0345 (15)	0.0042 (15)	0.0166 (16)	0.0019 (11)
O2	0.0513 (15)	0.0138 (12)	0.0359 (13)	0.0002 (9)	0.0113 (11)	−0.0005 (9)
O3	0.0333 (14)	0.057 (2)	0.087 (3)	−0.0027 (12)	0.0096 (15)	−0.0362 (16)
O4	0.0545 (16)	0.0235 (14)	0.0279 (13)	0.0028 (10)	0.0050 (11)	−0.0025 (8)
O5	0.122 (5)	0.088 (4)	0.175 (6)	−0.026 (4)	−0.030 (4)	0.009 (4)
O6	0.107 (3)	0.071 (3)	0.193 (6)	−0.016 (3)	0.076 (4)	−0.010 (3)
O7	0.100 (3)	0.085 (3)	0.081 (2)	−0.040 (2)	0.052 (2)	−0.047 (2)
C1	0.0404 (18)	0.0200 (18)	0.0345 (19)	0.0026 (13)	0.0053 (14)	0.0001 (13)
C2	0.045 (2)	0.0178 (16)	0.0322 (17)	0.0039 (14)	0.0096 (14)	0.0022 (15)
C3	0.047 (2)	0.025 (2)	0.067 (3)	−0.0066 (15)	0.027 (2)	−0.0025 (17)
C4	0.049 (2)	0.0226 (19)	0.064 (3)	0.0014 (16)	0.0288 (19)	−0.0039 (18)
C5	0.048 (3)	0.108 (5)	0.175 (7)	−0.011 (3)	0.018 (4)	−0.102 (5)
C6	0.044 (3)	0.075 (4)	0.179 (7)	−0.001 (3)	0.006 (3)	−0.077 (4)
C8	0.080 (4)	0.080 (4)	0.085 (4)	0.009 (3)	0.016 (3)	−0.005 (3)
C9	0.093 (5)	0.090 (5)	0.159 (8)	0.012 (4)	0.053 (5)	−0.018 (5)
C10	0.061 (3)	0.154 (8)	0.112 (6)	0.011 (4)	−0.004 (3)	0.021 (6)
F1	0.0747 (18)	0.0350 (15)	0.175 (4)	−0.0207 (14)	0.075 (2)	−0.0258 (18)
F2	0.0752 (19)	0.0419 (15)	0.157 (3)	−0.0095 (13)	0.076 (2)	−0.0325 (17)

### Geometric parameters (Å, °)

**Table:**

Cu1—O4	1.962 (3)	C3—F1	1.344 (4)
Cu1—O4^i^	1.962 (3)	C3—C4^ii^	1.382 (6)
Cu1—O2^i^	1.971 (3)	C4—F2	1.343 (4)
Cu1—O2	1.971 (3)	C4—C3^ii^	1.382 (6)
Cu1—O3	2.444 (3)	C5—C6^iii^	1.355 (8)
O1—C1	1.234 (4)	C5—H5A	0.9700
O2—C1	1.259 (4)	C5—H5B	0.9700
O3—C5	1.361 (6)	C6—C5^iii^	1.355 (8)
O3—C6	1.409 (6)	C6—H6A	0.9700
O4—H4A	0.8203	C6—H6B	0.9700
O4—H4B	0.8202	C7—C8	1.424 (9)
O5—C7	1.381 (9)	C7—H7A	0.9700
O5—C10	1.412 (9)	C7—H7B	0.9700
O6—C8	1.405 (7)	C8—H8A	0.9700
O6—C9	1.411 (8)	C8—H8B	0.9700
O7—H7C	0.8202	C9—C10	1.496 (11)
O7—H7D	0.8203	C9—H9A	0.9700
C1—C2	1.515 (5)	C9—H9B	0.9700
C2—C3	1.372 (5)	C10—H10A	0.9700
C2—C4	1.382 (5)	C10—H10B	0.9700
			
O4—Cu1—O4^i^	180.0	O3—C5—H5A	107.0
O4—Cu1—O2^i^	93.24 (8)	C6^iii^—C5—H5B	107.0
O4^i^—Cu1—O2^i^	86.76 (8)	O3—C5—H5B	107.0
O4—Cu1—O2	86.76 (8)	H5A—C5—H5B	106.8
O4^i^—Cu1—O2	93.24 (8)	C5^iii^—C6—O3	118.4 (5)
O2^i^—Cu1—O2	180.0	C5^iii^—C6—H6A	107.7
O4—Cu1—O3	91.16 (13)	O3—C6—H6A	107.7
O4^i^—Cu1—O3	88.85 (13)	C5^iii^—C6—H6B	107.7
O2^i^—Cu1—O3	90.79 (9)	O3—C6—H6B	107.7
O2—Cu1—O3	89.21 (9)	H6A—C6—H6B	107.1
C1—O2—Cu1	129.4 (2)	O5—C7—C8	112.9 (6)
C5—O3—C6	114.3 (4)	O5—C7—H7A	109.0
C5—O3—Cu1	124.9 (3)	C8—C7—H7A	109.0
C6—O3—Cu1	120.7 (3)	O5—C7—H7B	109.0
Cu1—O4—H4A	103.2	C8—C7—H7B	109.0
Cu1—O4—H4B	115.3	H7A—C7—H7B	107.8
H4A—O4—H4B	121.3	O6—C8—C7	111.1 (6)
C7—O5—C10	112.2 (6)	O6—C8—H8A	109.4
C8—O6—C9	110.2 (5)	C7—C8—H8A	109.4
H7C—O7—H7D	121.4	O6—C8—H8B	109.4
O1—C1—O2	127.2 (3)	C7—C8—H8B	109.4
O1—C1—C2	117.3 (3)	H8A—C8—H8B	108.0
O2—C1—C2	115.5 (3)	O6—C9—C10	109.0 (6)
C3—C2—C4	115.8 (3)	O6—C9—H9A	109.9
C3—C2—C1	122.6 (3)	C10—C9—H9A	109.9
C4—C2—C1	121.6 (3)	O6—C9—H9B	109.9
F1—C3—C2	119.0 (3)	C10—C9—H9B	109.9
F1—C3—C4^ii^	118.7 (3)	H9A—C9—H9B	108.3
C2—C3—C4^ii^	122.2 (3)	O5—C10—C9	109.7 (5)
F2—C4—C3^ii^	118.9 (3)	O5—C10—H10A	109.7
F2—C4—C2	119.1 (3)	C9—C10—H10A	109.7
C3^ii^—C4—C2	122.0 (3)	O5—C10—H10B	109.7
C6^iii^—C5—O3	121.3 (5)	C9—C10—H10B	109.7
C6^iii^—C5—H5A	107.0	H10A—C10—H10B	108.2
			
O4—Cu1—O2—C1	166.7 (3)	C1—C2—C3—F1	−1.6 (6)
O4^i^—Cu1—O2—C1	−13.3 (3)	C4—C2—C3—C4^ii^	−1.1 (7)
O3—Cu1—O2—C1	−102.1 (3)	C1—C2—C3—C4^ii^	178.9 (4)
O4—Cu1—O3—C5	55.5 (6)	C3—C2—C4—F2	178.8 (4)
O4^i^—Cu1—O3—C5	−124.5 (6)	C1—C2—C4—F2	−1.2 (6)
O2^i^—Cu1—O3—C5	148.8 (6)	C3—C2—C4—C3^ii^	1.1 (7)
O2—Cu1—O3—C5	−31.2 (6)	C1—C2—C4—C3^ii^	−178.9 (4)
O4—Cu1—O3—C6	−123.5 (5)	C6—O3—C5—C6^iii^	−27.8 (12)
O4^i^—Cu1—O3—C6	56.5 (5)	Cu1—O3—C5—C6^iii^	153.1 (6)
O2^i^—Cu1—O3—C6	−30.2 (5)	C5—O3—C6—C5^iii^	26.9 (12)
O2—Cu1—O3—C6	149.8 (5)	Cu1—O3—C6—C5^iii^	−154.0 (6)
Cu1—O2—C1—O1	2.5 (6)	C10—O5—C7—C8	54.0 (9)
Cu1—O2—C1—C2	−176.9 (2)	C9—O6—C8—C7	58.4 (8)
O1—C1—C2—C3	115.0 (4)	O5—C7—C8—O6	−55.3 (9)
O2—C1—C2—C3	−65.5 (5)	C8—O6—C9—C10	−59.1 (9)
O1—C1—C2—C4	−65.0 (5)	C7—O5—C10—C9	−54.3 (9)
O2—C1—C2—C4	114.5 (4)	O6—C9—C10—O5	57.0 (10)
C4—C2—C3—F1	178.4 (4)		

Symmetry codes: (i) −*x*+1, −*y*, −*z*; (ii) −*x*+1, −*y*+1, −*z*; (iii) −*x*, −*y*, −*z*.

### Hydrogen-bond geometry (Å, °)

**Table:**

*D*—H···*A*	*D*—H	H···*A*	*D*···*A*	*D*—H···*A*
O4—H4B···O7	0.82	1.92	2.670 (5)	152
C5—H5B···F1	0.97	2.53	3.453 (8)	160
O4—H4A···O1^i^	0.82	1.85	2.641 (3)	162
O7—H7C···O6^iv^	0.82	2.03	2.807 (7)	158
O7—H7D···O1^v^	0.82	2.09	2.797 (5)	144

Symmetry codes: (i) −*x*+1, −*y*, −*z*; (iv) *x*, *y*−1, *z*; (v) *x*, −*y*+1/2, *z*+1/2.
